# Majorana flat band edge modes of topological gapless phase in 2D Kitaev square lattice

**DOI:** 10.1038/s41598-019-41529-y

**Published:** 2019-03-21

**Authors:** K. L. Zhang, P. Wang, Z. Song

**Affiliations:** School of Physics, Nankai University, Tianjin, 300071 China

## Abstract

We study a Kitaev model on a square lattice, which describes topologically trivial superconductor when gap opens, while supports topological gapless phase when gap closes. The degeneracy points are characterized by two vortices in momentum space, with opposite winding numbers. We show rigorously that the topological gapless phase always hosts a partial Majorana flat band edge modes in a ribbon geometry, although such a single band model has zero Chern number as a topologically trivial superconductor. The flat band disappears when the gapless phase becomes topologically trivial, associating with the mergence of two vortices. Numerical simulation indicates that the flat band is robust against the disorder.

## Introduction

Topological materials have become the focus of intense research in the last years^1–4^, since they not only exhibit new physical phenomena with potential technological applications, but also provide a fertile ground for the discovery of fermionic particles and phenomena predicted in high-energy physics, including Majorana^5–10^, Dirac^11–17^ and Weyl fermions^18–26^. These concepts relate to Majorana edge modes and topological gapless phases. System in the topological gapless phase exhibits band structures with band-touching points in the momentum space, where these kinds of nodal points appear as topological defects of an auxiliary vector field. On the other hand, a gapful phase can be topologically non-trivial, commonly referred to as topological insulators and superconductors, the band structure of which is characterized by nontrivial topology. A particularly important concept is the bulk-edge correspondence, which links the nontrivial topological invariant in the bulk to the localized edge modes. The number of Majorana edge modes is determined by bulk topological invariant. In general, edge states are the eigenstates of Hamiltonian that are exponentially localized at the boundary of the system. The Majorana edge modes have been actively pursued in condensed matter physics^27–33^ since spatially separated Majorana fermions lead to degenerate ground states, which encode qubits immune to local decoherence^34^. This bulk-edge correspondence indicates that a single-band model must have vanishing Chern number and there should be no edge modes when open boundary conditions are applied. However, the existence of topological gapless indicates that there is hidden topological feature in some single band system. A typical system is a 2D honeycomb lattice of been graphene, which is a zero-band-gap semiconductor with a linear dispersion near the Dirac point. Meanwhile, there is another interesting feature lies in the appearance of partial flat band edge modes in a ribbon geometry^35–37^, which exhibit robustness against disorder^38^. Recently, it has been pointed that Majorana zero modes are not only attributed to topological superconductors. A 2D topologically trivial superconductors without chiral edge modes can host robust Majorana zero modes in topological defects^39–41^.

In this paper, we investigate this issue through an exact solution of a concrete system. We study a Kitaev model on a square lattice, which describes topologically trivial superconductor when gap opens, while supports topological gapless phase when gap closes^42,43^. The degeneracy points are characterized by two vortices, or Dirac nodal points in momentum space, with opposite winding numbers. This work aims to shed light on the nature of topological edge modes associated with topological gapless phase, rather than gapful topological superconductor. We show rigorously that the topological gapless phase always hosts a partial Majorana flat band edge modes in a ribbon geometry. The flat band disappears when the gapless phase becomes topologically trivial, associating with the mergence of two vortices. Numerical simulation indicates that the flat band is robust against the disorder.

## Results

We have demonstrated that a topologically trivial superconductor emerges as a topological gapless state, which support Majorana flat band edge modes. The new quantum state is characterized by two linear band-degeneracy points with opposite topological invariant. In sharp contrast to the conventional topological superconductor, such a system has single band, thus has zero Chern number. We prove that the appearance of this topological feature attributes to the corresponding Majorana lattice structure, which is a modified honeycomb lattice. It is natural to acquire a set of zero modes, which is robust against disorder. In the following, there are three parts: (i) We present the Kitaev Hamiltonian on a square lattice and the phase diagram for the topological gapless phase. (ii) We investigate the Majorana bound states. (iii) We perform numerical simulation to investigate the robust of the edge modes against the disorder perturbations.

### Model and topological gapless phase

We consider the Kitaev model on a square lattice which is employed to depict 2D *p*-wave superconductors. The Hamiltonian of the tight-binding model reads

1 $$\begin{array}{c}H=-\,t\,{\sum }_{{\bf{r}},{\bf{a}}}{c}_{{\bf{r}}}^{\dagger }{c}_{{\bf{r}}+{\bf{a}}}-{\rm{\Delta }}\,{\sum }_{{\bf{r}},{\bf{a}}}{c}_{{\bf{r}}}{c}_{{\bf{r}}+{\bf{a}}}+{\rm{h}}{\rm{.c}}{\rm{.}}\\ \,\,+\mu \,{\sum }_{{\bf{r}}}(2{c}_{{\bf{r}}}^{\dagger }{c}_{{\bf{r}}}-1),\end{array}$$

where **r** is the coordinates of lattice sites and *c*_**r**_ is the fermion annihilation operators at site **r**. Vectors **a** = *a***i**, *a***j**, are the lattice vectors in the *x* and *y* directions with unitary vectors **i** and **j**. The hopping between neighboring sites is described by the hopping amplitude *t*. The isotropic order parameter Δ is real, which result in topologically trivial superconductor. The last term gives the chemical potential.

Taking the Fourier transformation

2 $${c}_{{\bf{r}}}=\frac{1}{N}\,{\sum }_{{\bf{k}}}{c}_{{\bf{k}}}{e}^{i{\bf{k}}\cdot {\bf{r}}},$$

the Hamiltonian with periodic boundary conditions on both directions can be rewritten as

3 $$H={\sum }_{{\bf{k}}}({c}_{{\bf{k}}}^{\dagger }\,{c}_{-{\bf{k}}}){h}_{{\bf{k}}}(\begin{array}{c}{c}_{{\bf{k}}}\\ {c}_{-\,{\bf{k}}}^{\dagger }\end{array}),$$

where

4 $${h}_{{\bf{k}}}=(\begin{array}{cc}\mu -t\,\cos \,{k}_{x}-t\,\cos \,{k}_{y} & i{\rm{\Delta }}(\sin \,{k}_{x}+\,\sin \,{k}_{y})\\ -i{\rm{\Delta }}(\sin \,{k}_{x}+\,\sin \,{k}_{y}) & -\mu +t\,\cos \,{k}_{x}+t\,\cos \,{k}_{y}\end{array}),$$

where the summation of **k** = (*k*_*x*_, *k*_*y*_) is ${\sum }_{{\bf{k}}}={\sum }_{{k}_{x}=-\pi }^{\pi }{\sum }_{{k}_{y}=-\pi }^{\pi }$. The core matrix can be expressed as

5 $${h}_{{\bf{k}}}={\bf{B}}({\bf{k}})\cdot \sigma ,$$

where the components of the auxiliary field **B**(**k**) = (*B*_*x*_, *B*_*y*_, *B*_*z*_) are

6 $$\{\begin{array}{rcl}{B}_{x} & = & 0\\ {B}_{y} & = & -{\rm{\Delta }}(\sin \,{k}_{x}+\,\sin \,{k}_{y})\\ {B}_{z} & = & \mu -t\,\cos \,{k}_{x}-t\,\cos \,{k}_{y}\end{array}.$$

σ are the Pauli matrices

7 $${\sigma }_{x}=(\begin{array}{cc}0 & 1\\ 1 & 0\end{array}),{\sigma }_{y}=(\begin{array}{cc}0 & -i\\ i & 0\end{array}),{\sigma }_{z}=(\begin{array}{cc}1 & 0\\ 0 & -1\end{array})\mathrm{.}$$

**Figure 1:**
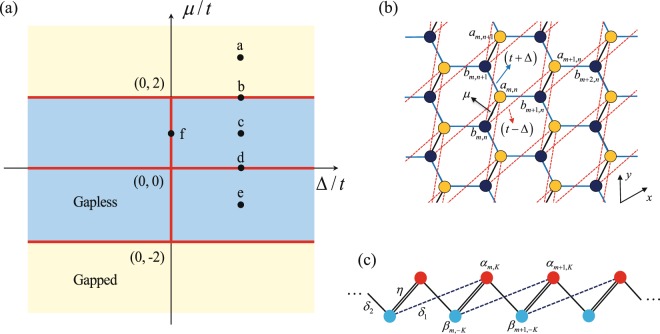
(**a**) Phase diagram of the Kitaev model on a square lattice system on the parameter *μ* − Δ plane (in units of *t*). The red lines indicate the boundary, which separate the topologically trivial gapped phases (yellow) and topological gapless phases (blue). The system at the boundary (red lines) is topologically trivial gapless phase. (**b**) Schematically illustration of the Majorana lattice, which is honeycomb geometry with long-range hopping term. (**c**) The geometry of the auxiliary operator lattice represented in Eq. (22), which is an SSH chain with long-range hopping term. The edge modes of a set of modified SSH chains form Majorana fat band edge modes in (**b**) when the system is in the blue region of the phase diagram (**a**).

The parameters *t*, Δ and *μ* are real number as illustrated in the phase diagram (Fig. 1(a)), which automatically requires *B*_*x*_ = 0. The Bogoliubov spectrum is

8 $${\varepsilon }_{{\bf{k}}}=|{\bf{B}}({\bf{k}})|=2\sqrt{{[\mu -t(\cos {k}_{x}+\cos {k}_{y})]}^{2}+{{\rm{\Delta }}}^{2}{(\sin {k}_{x}+\sin {k}_{y})}^{2}}.$$

We are interested in the gapless state arising from the band touching point of the spectrum. The band degenerate point **k**_0_ = (*k*_0*x*_, *k*_0*y*_) is determined by

9 $$\{\begin{array}{lcl}-{\rm{\Delta }}(\sin \,{k}_{0x}+\,\sin \,{k}_{0y}) & = & 0,\\ \mu -t(\cos \,{k}_{0x}+\,\cos \,{k}_{0y}) & = & 0.\end{array}$$

**Figure 2:**
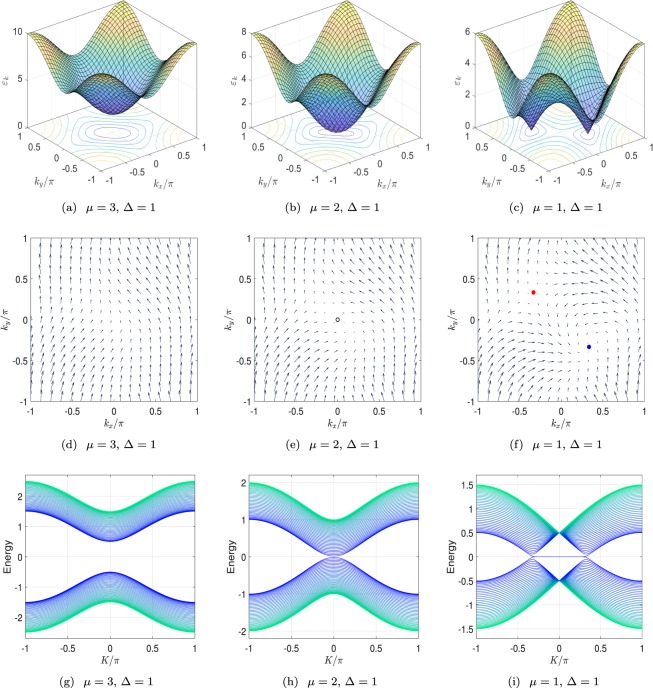
(**a**–**c**) Plots of energy spectra from Eq. (8) at three typical points a, b, and c marked in the phase diagram in Fig. 1. There is gapped in (**a**), a single degeneracy point with parabolic dispersion in (**b**), and two degeneracy points with linear dispersion in (**c**). (**d**–**f**) Plots of field defined in Eq. (6) in the momentum space for three cases corresponding to (**a**–**c**). There are two vortices in (**f**) with opposite winding numbers ±1. As *μ* increases, two vortices close and merge into a single point in (**e**). As *μ* increases to 3, the field become trivial in (**d**). (**g**–**i**) Plots of the spectra from Eq. (21) with *M* = 40 for three cases of (**a**–**c**). It indicates that the existence of pair of vortices links to a flat band of Majorana lattice.

**Figure 3:**
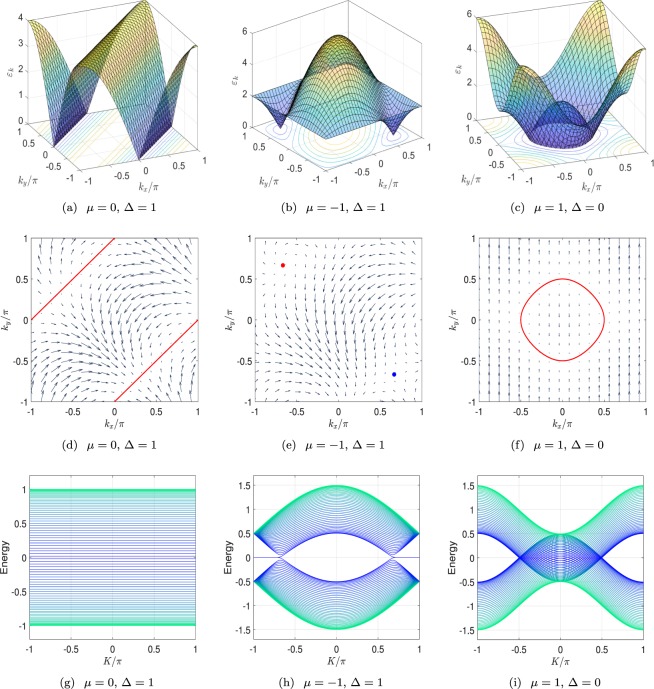
(**a**–**c**) Plots of energy spectra from Eq. (8) at three typical points d, e, and f marked in the phase diagram in Fig. 1. There is two degeneracy lines in (**a**), two degeneracy points with linear dispersion in (**b**), and a degeneracy loop in (**c**). (**d**–**f**) Plots of field defined in Eq. (6) in the momentum space for three cases corresponding to (**a**–**c**). There are two vortices in (**e**) with opposite winding numbers ±1. (**g**–**i**) Plots of the spectra from Eq. (21) with *M* = 40 for three cases of (**a**–**c**). It indicates that the existence of pair of vortices links to a flat band of Majorana lattice.

As pointed in ref.^42^, two bands touch at three types of configurations: single point, double points, and curves in the *k*_*x*_ − *k*_*y*_ plane, determined by the region of parameter Δ−*μ* plane (in units of *t*). We focus on the non-trivial case with nonzero Δ. Then we have

10 $${k}_{0x}=-\,{k}_{0y}=\pm \arccos (\frac{\mu }{2t})$$

in the region $|\mu /t|\le 2$, which indicates that there are two nodal points for *μ* ≠ 0 and |*μ*/*t*| ≠ 2. The two points move along the line represented by the equation *k*_0*x*_ = −*k*_0*y*_, and merge at **k**_0_ = (*π*, −*π*) when *μ*/*t* = ±2. In the case of *μ* = 0, the nodal points become two nodal lines represented by the equations *k*_0*y*_ = ±*π* + *k*_0*x*_. The phase diagram is illustrated in Fig. 1, depending on the values of *μ* and Δ (compared with the hopping strength *t*). We plot the band structures in Figs 2 and 3 for several typical cases.

In the vicinity of the degeneracy points, we have

11 $$\{\begin{array}{rcl}{B}_{x} & = & 0\\ {B}_{y} & = & -{\rm{\Delta }}\,\cos \,{k}_{0x}({q}_{y}+{q}_{x})\\ {B}_{z} & = & t\,\sin \,{k}_{0x}({q}_{x}-{q}_{y})\end{array},$$

where **q** = **k** − **k**_0_, **k**_0_ = (*k*_0*x*_, *k*_0*y*_) and (*k*_0*x*_, *k*_0*y*_) satisfy Eq. (10), is the momentum in another frame. Around these degeneracy points, the Hamiltonian *h*_k_ can be linearized as the form

12 $$ {\mathcal H} ({\bf{q}})={\sum }_{i,j}{a}_{ij}{q}_{i}{\sigma }_{j},$$

which is equivalent to the Hamiltonian for two-dimensional massless relativistic fermions. Here (*q*_1_, *q*_2_) = (*q*_*x*_, *q*_*y*_) and (*σ*_1_, *σ*_2_) = (*σ*_*y*_, *σ*_*z*_). The corresponding chirality for these particle is defined as

13 $$w={\rm{sgn}}[{\rm{\det }}({a}_{ij}\mathrm{)].}$$

Then we have

14 $${\rm{\det }}|\begin{array}{cc}-{\rm{\Delta }}\,\cos \,{k}_{0x} & t\,\sin \,{k}_{0x}\\ -{\rm{\Delta }}\,\cos \,{k}_{0x} & -t\,\sin \,{k}_{0x}\end{array}|=t{\rm{\Delta }}\,\sin \,2{k}_{0x},$$

which leads to *w* = ±1 for two nodal points. The chiral relativistic fermions serve as two-dimensional Dirac fermions. Two Dirac nodes located at two separated degenerate points have opposite chirality. We note that for Δ|*μ*/*t*|(|*μ*/*t*| − 2) = 0, we have *w* = 0. At this situation, two Dirac nodes merge at (0, 0) and (±*π*, ∓*π*). The topology of the nodal point becomes trivial, and a perturbation hence can open up the energy gap. We illustrate the vortex structure of the degeneracy point in *k*_*x*_ − *k*_*y*_ plane in Figs 2 and 3. As shown in figures, we find three types of topological configurations: pair of vortices with opposite chirality, single trivial vortex (or degeneracy lines), and no vortex, corresponding to topological gapless, trivial gapless and gapped phases, respectively.

### Majorana flat band edge modes

Now we turn to study the feature of gapless phase in the framework of Majorana representation. The Kitaev model on a honeycomb lattice and chain provides well-known examples of systems with such a bulk-boundary correspondence^44–50^. It is well known that a sufficient long chain has Majorana modes at its two ends^51^. A number of experimental realizations of such models have found evidence for such Majorana modes^7,52–55^. In contrast to previous studies based on a gapful system with nonzero Chern number, we focus on the Kitaev model in the topologically trivial phase. This is motivated by the desire to get a connection between the Majorana edge modes and topological nature hidden in a topologically trivial superconductor. At first, we revisit the description of the present model on a cylindrical lattice in terms of Majorana fermions.

We introduce Majorana fermion operators

15 $${a}_{{\bf{r}}}={c}_{{\bf{r}}}^{\dagger }+{c}_{{\bf{r}}},{b}_{{\bf{r}}}=-\,i({c}_{{\bf{r}}}^{\dagger }-{c}_{{\bf{r}}}),$$

which satisfy the relations

16 $$\begin{array}{rcl}\{{a}_{{\bf{r}}},{a}_{{\bf{r}}^{\prime} }\} & = & 2{\delta }_{{\bf{r}},{\bf{r}}^{\prime} },\{{b}_{{\bf{r}}},{b}_{{\bf{r}}^{\prime} }\}=2{\delta }_{{\bf{r}},{\bf{r}}^{\prime} },\\ \{{a}_{{\bf{r}}},{b}_{{\bf{r}}^{\prime} }\} & = & \mathrm{0,}{a}_{{\bf{r}}}^{2}={b}_{{\bf{r}}}^{2}=1.\end{array}$$

Then the Majorana representation of the Hamiltonian is

17 $$H=\frac{1}{4}\,{\sum }_{{\bf{r}}}[i(t+{\rm{\Delta }})\,{\sum }_{{\bf{a}}}{a}_{{\bf{r}}}{b}_{{\bf{r}}+{\bf{a}}}+\,i(t-{\rm{\Delta }}){\sum }_{{\bf{a}}}{a}_{{\bf{r}}+{\bf{a}}}{b}_{{\bf{r}}}-i2\mu {a}_{{\bf{r}}}{b}_{{\bf{r}}}+{\rm{h}}{\rm{.c}}\mathrm{.].}$$

It represents a honeycomb lattice with extra hopping term *a*_r+a_*b*_r_, which is schematically illustrated in Fig. 1. Before a general investigation, we consider a simple case to show that a flat band Majorana modes do exist. Taking *t* = Δ = *μ* the Hamiltonian reduces to

18 $${H}_{{\rm{hc}}}=\frac{t}{2}\,{\sum }_{{\bf{r}}}(i{a}_{{\bf{r}}}{\sum }_{{\bf{a}}}{b}_{{\bf{r}}+{\bf{a}}}-i{a}_{{\bf{r}}}{b}_{{\bf{r}}}+{\rm{h}}{\rm{.c}}\mathrm{.)}\mathrm{.}$$

which corresponds to a honeycomb ribbon with zigzag boundary condition. It is well-known that there exist a partial flat band edge modes in such a lattice system^35–38^.

In the following, we will show that this feature still remains in a wide parameter region. Consider the lattice system on a cylindrical geometry by taking the periodic boundary condition in one direction and open boundary in another direction. For a *M* × *M* Kitaev model, the Majorana Hamiltonian can be explicitly expressed as

19 $$\begin{array}{rcl}{H}_{{\rm{M}}} & = & \frac{i}{4}\,{\sum }_{l,j=1}^{M}[(t-{\rm{\Delta }})({a}_{m,n+1}{b}_{m,n}+{a}_{m+\mathrm{1,}n}{b}_{m,n})\\  &  & +\,(t+{\rm{\Delta }})({a}_{m,n}{b}_{m,n+1}+{a}_{m,n}{b}_{m+\mathrm{1,}n})-2\mu {a}_{m,n}{b}_{m,n}-{\rm{h}}{\rm{.c}}\mathrm{.]},\end{array}$$

by taking **r** = *m***i** + *n***j** → (*m*, *n*). The boundary conditions are *a*_*n*,1_ = *a*_*n*,*M*+1_, *b*_*n*,1_ = *b*_*n*,*M*+1_, *a*_*M*+1,*n*_ = 0, and *b*_*M*+1,*n*_ = 0.

Consider the Fourier transformations of Majorana operators

20 $$\{\begin{array}{c}{\alpha }_{m,K}=\frac{1}{\sqrt{N}}\,{\sum }_{n=1}^{M}{e}^{-iKn}{a}_{m,n}\\ {\beta }_{m,K}=\frac{1}{\sqrt{N}}\,{\sum }_{n=1}^{M}{e}^{-iKn}{b}_{m,n}\end{array},$$

where the wave vector *K* = 2*πl*/*N*, *l* = 1, 2, …, *N*.

The Hamiltonian *H*_M_ can be rewritten as

21 $${H}_{{\rm{M}}}={\sum }_{K}{h}_{{\rm{M}}}^{K},$$

22 $$\begin{array}{rcl}{h}_{{\rm{M}}}^{K} & = & {\sum }_{m=1}^{M-1}(\eta {\alpha }_{m,K}i{\beta }_{m,-K}+{\delta }_{1}{\alpha }_{m+1,K}i{\beta }_{m,-K}\\  &  & +{\delta }_{2}{\alpha }_{m,K}i{\beta }_{m+1,-K})+\eta {\alpha }_{M,K}i{\beta }_{M,-K}+{\rm{h}}{\rm{.c}}.,\end{array}$$

where *η* = [(*t* − Δ)*e*^*iK*^ + (*t* + Δ)*e*^−*iK*^ − 2*μ*]/4, *δ*_1_ = (*t* − Δ)/4 and *δ*_2_ = (*t* + Δ)/4, and ${h}_{{\rm{M}}}^{K}$ obeys

23 $${h}_{{\rm{M}}}^{K},{h}_{{\rm{M}}}^{K^{\prime} }]=\mathrm{0,}$$

i.e., *H*_M_ has been block diagonalized. We would like to point that operators *α*_*m*,*K*_ and *β*_*m*,*K*_ are not Majorana fermion operators except the cases with *K* = 0 or *π*. We refer such operators as to auxiliary operators. We note that each ${h}_{{\rm{M}}}^{K}$ represents a modified SSH chain about auxiliary operators *α*_*m*,*K*_ and *β*_*m*,*K*_ with *η*, *δ*_1_, and *δ*_2_ hopping terms. One can always get a diagonalized ${h}_{{\rm{M}}}^{K}$ through the diagonalization of the matrix of the corresponding single-particle modified SSH chain. For simplicity, we only consider the case with positive parameters *t*, Δ, and *μ*. In large ***M*** limit, there are two zero modes for ${h}_{{\rm{M}}}^{K}$ under the condition 0 < *μ* < 2 (in units of *t*). Actually, it can be checked that ${h}_{{\rm{M}}}^{K}$ can contribute a term

24 $$0\times ({\gamma }_{K}^{\dagger }{\gamma }_{K}-{\gamma }_{K}{\gamma }_{K}^{\dagger }),$$

where

25 $${\gamma }_{K}=A\,{\sum }_{j=1}^{M}[({p}_{+}^{M-j+1}-{p}_{-}^{M-j+1}){\alpha }_{j,K}+i({p}_{+}^{j}-{p}_{-}^{j}){\beta }_{j,K}].$$

Here $A={(2\sum _{j=1}^{M}{|{p}_{+}^{j}-{p}_{-}^{j}|}^{2})}^{-\frac{1}{2}}$ is normalization constant, and

26 $${p}_{\pm }=\frac{\pm \sqrt{{|\eta |}^{2}-4{\delta }_{1}{\delta }_{2}}-|\eta |}{2{\delta }_{2}}.$$

The term in Eq. (24) exists under the convergence condition

27 $$\mathop{\mathrm{lim}}\limits_{j\to \infty }({p}_{+}^{j}-{p}_{-}^{j})=0.$$

As expected, we note that operators *γ*_*K*_ satisfy the fermion commutation relations

28 $$({\gamma }_{K},{\gamma }_{K^{\prime} }^{\dagger })=2{\delta }_{KK^{\prime} },({\gamma }_{K},{\gamma }_{K^{\prime} })=0,$$

representing edge modes. The sufficient condition for Eq. (27) is

29 $$|{p}_{\pm }| < 1,$$

which leads to

30 $$\{\begin{array}{l}|\eta | < 2|{\delta }_{2}|\\ -{|{\delta }_{2}|}^{2}+|\eta {\delta }_{2}| < {\delta }_{1}{\delta }_{2}\le {|\frac{\eta }{2}|}^{2}\end{array},$$

or more explicitly form

31 $$\{\begin{array}{l}R-2t{\rm{\Delta }} < 0\\ {{\rm{\Delta }}}^{2} > R+2{{\rm{\Delta }}}^{2}\ge 0\end{array},$$

where $R(K,{\rm{\Delta }},\mu ,t)=({t}^{2}-{{\rm{\Delta }}}^{2})\,{\cos }^{{\rm{2}}}(K)-2\mu t\,\cos (K)+{\mu }^{2}-{t}^{2}$. To demonstrate this result, considering a simple case with Δ = *μ* = *t*, we find that ***R*** reduces to −2Δ^2^cos(*K*) and satisfies above equations when take −*π*/3 < *K* < *π*/3. Furthermore the parameters become *η* = Δ(*e*^−*iK*^ − 1)/2, *δ*_1_ = 0 and *δ*_2_ = Δ/2, and ${h}_{{\rm{M}}}^{K}$ corresponds to a simple SSH chain with |*η*| < |*δ*_2_|. The edge mode wave functions can be obtained from *p*_+_ = 0 and ${p}_{-}=-\,\sqrt{2(1-\,\cos \,K)}$. Then the existence of edge modes is well reasonable. The zero modes in the plot of energy band in Fig. 2(i) corresponds this flat band of edge mode.

### Disorder perturbation

One of the most striking features of topologically protected edge states is the robustness against to certain types of disorder perturbation to the original Hamiltonian. In this section, we investigate the robustness of the Majorana edge flat band in the presence of disorder. The disorder we discuss here arises from the parameters {*t*, Δ, *μ*} in the Hamiltonian *H*_M_. More precisely, one can rewrite the Hamiltonian in the form

32 $${H}_{{\rm{M}}}={\psi }^{\dagger }h\psi ,$$

where *h* represents a 2*M*^2^ × 2*M*^2^ matrix in the basis

33 $$\begin{array}{rcl}\psi  & = & ({a}_{1,1},i{b}_{1,1},\mathrm{...},{a}_{1,M},i{b}_{1,M},\\  &  & {a}_{2,1},i{b}_{2,1},\mathrm{...},{a}_{2,M},i{b}_{2,M},\\  &  & \mathrm{...},{a}_{i,j},i{b}_{i,j},\mathrm{...},\\  &  & {a}_{M,1},i{b}_{M,1},\mathrm{...},{a}_{M,M},i{b}_{M,M}{)}^{T}.\end{array}$$

We introduce the disorder perturbation to *H*_M_ by preserving the time reversal symmetry, i.e., keeping the reality of the parameters {*t*, Δ, *μ*}. We take the randomized matrix elements in *h* by the replacement

34 $$\{\begin{array}{rcl}t & \to  & {t}_{l.j}={r}_{l,j}^{a}t\\ {\rm{\Delta }} & \to  & {{\rm{\Delta }}}_{l.j}={r}_{l,j}^{b}{\rm{\Delta }}\\ \mu  & \to  & {\mu }_{l.j}={r}_{l,j}^{c}\mu \end{array},$$

to get the disorder matrix *h*_DP_. Here *r*^*a*,*b*,*c*^ are three ***M*** × ***M*** matrices which consist of random numbers in the interval of (1 − *ξ*, 1 + *ξ*), influencing each matrix elements. Real factor *ξ* plays the role of the disorder strength.

**Figure 4:**
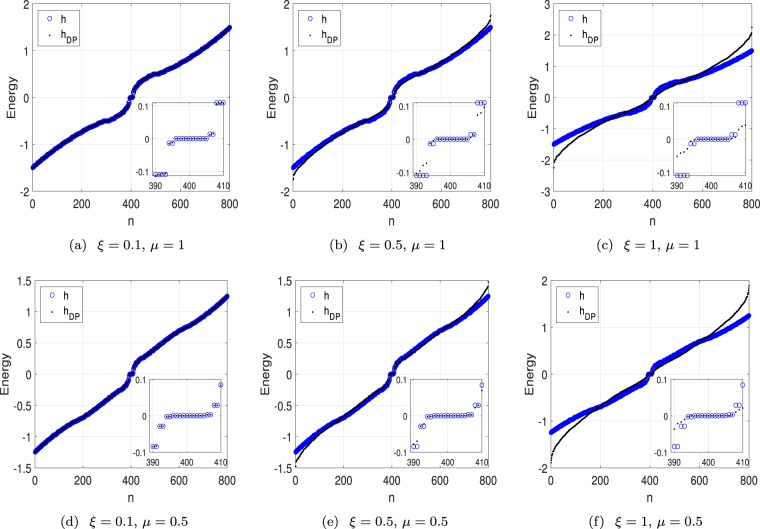
Plots of eigenvalues of matrices *h* and *h*_DP_ with typical parameters and different disorder strength factor *ξ*. The dimension of matrix is 800, corresponding to *M* = 20. According to analytical analysis, there are 10 and 14 quasi-zero modes in the upper and lower panels. Here we take Δ = *t* = 1. Numerical results show that as *ξ* increases, most of levels of *h*_DP_ deviate from that of *h*, while the zero-mode levels remain unchanged, indicating the robustness of zero modes against to the disorder.

We investigate the influence of nonzero *ξ* by comparing two sets of eigenvalues obtained by numerical diagonalization of finite-dimensional matrices *h* and *h*_DP_, respectively. The plots in Fig. 4. indicate that the zero modes remain unchanged in the presence of random perturbations with not too large *ξ*. The numerical result support our conclusion that the topological gapless states correspond to the presence of topologically protected edge modes.

## Discussion

According to the bulk-edge correspondence, it seems that the existence of edge states requires a gapped topological phase. This may not include the case with a single band which contains topological gapless states. The topological character of a gapless state does not require the existence of the gap. This arises the question: What is the essential reason for the edge state, energy gap or topology of the energy band? Obviously, energy gap is not since many gapped systems do not support the edge states. Then it is possible that a special single band system supports the edge states. In the case of the lack of an exact proof, concrete example is desirable. As such an example we have considered a Kitaev model on a square lattice, which describes topologically trivial superconductor when gap opens, while supports topological gapless phase when gap closes. The degeneracy points are characterized by two vortices, or Dirac nodal points in momentum space, with opposite winding numbers. We demonstrated that a topologically trivial superconductor emerges as a topological gapless state, which support Majorana flat band edge modes. The new quantum state is characterized by two linear band-degeneracy points with opposite topological invariant. In sharp contrast to the conventional topological superconductor, such a system has single band, thus has zero Chern number. We prove that the appearance of this topological feature attributes to the corresponding Majorana lattice structure, which is a modified honeycomb lattice. The topological feature of an edge state is the robustness against disorder. The numerical results indicate that such a criteria is met for this concrete example. We also note that the topological gapless state and the edge state have the same energy level, which is also an open question in the future.
